# The effect of non-invasive positive airway pressure therapy following thoracic surgical procedures: protocol for a systematic review

**DOI:** 10.1186/s13643-015-0073-8

**Published:** 2015-06-12

**Authors:** Elinaldo da Conceição dos Santos, Adriana Claudia Lunardi

**Affiliations:** Master’s and Doctoral Programs in Physical Therapy, Universidade Cidade de São Paulo, Cesário Galeno Street, 448/475, Sao Paulo, 03071-000 Brazil; Department of Biological and Health Sciences, Universidade Federal do Amapá, Macapá, Brazil; Department of Physical Therapy, School of Medicine, Universidade de São Paulo, São Paulo, Brazil

**Keywords:** Respiratory therapy, Thoracic surgery, Lung function, Pulmonary volume, Oxygenation, Systematic review

## Abstract

**Background:**

Thoracic surgical procedures impair respiratory function, decreasing ventilation and oxygenation and increasing the risk of acute respiratory failure and pulmonary complications. To prevent these clinical repercussions, positive airway pressure therapy is widely used to increase pulmonary ventilation, decrease muscle overload, and ensure adequate oxygenation; however, the benefit of this therapy remains unclear.

**Methods/design:**

A systematic search of the literature including PubMed, CINAHL, AMED, PsycINFO, LILACS, Scielo, Scopus, PEDro, and the Cochrane Library will identify the randomized and quasi-randomized trials that used CPAP, Bilevel, or IPPB compared with a control without intervention, a sham treatment or other lung expansion techniques following thoracic surgical procedures. From these trials, we will extract data on a predefined list of outcomes, including oxygenation, ventilation, respiratory failure, pulmonary complications, and time of resolution of the clinical condition. The methodological quality of each trial included will be assessed using the PEDro scale. The strength of the recommendations will be summarized using the GRADE scale. Meta-analyses will be performed, if appropriate.

**Discussion:**

This review aims to promote greater knowledge regarding the efficiency of the use of non-invasive positive airway pressure on recovery of respiratory function and on prevention of pulmonary complications following thoracic surgical procedures. This review could help health professionals improve the care for patients undergoing thoracic surgical procedures.

**Systematic review registration:**

PROSPERO CRD42015019004

**Electronic supplementary material:**

The online version of this article (doi:10.1186/s13643-015-0073-8) contains supplementary material, which is available to authorized users.

## Background

Thoracic surgical procedures impair respiratory function, depending on the duration and type of procedure as well as on each patient’s clinical condition [1]. Longer and more aggressive procedures in patients with chronic pulmonary diseases and worse clinical conditions could result in patients being more susceptible to pulmonary complications [2, 3] including atelectasis, bronchospasms, pneumonia, need of mechanical ventilation, or death [4]. Other complications following thoracic surgical procedures include air leakage from pleural fistulae [5], which has an incidence of 4.5 to 20 % after pneumonectomy and 0.5 % after lobectomy [6]. These complications increase the length of hospital stays and the costs [7].

Non-invasive positive airway pressure therapy has been used to decrease complications following thoracic surgical procedures [8]. Positive airway pressure maintains oxygen and carbon dioxide in normal ranges with minimal ventilator usage [9]. Its primary indications include ventilatory overload, alterations of the chest wall and lung expansion, increase of airway resistance, prevention of respiratory failure, and improvement of cough efficacy [10].

The effect of therapy with non-invasive positive airway pressure has been studied in other types of procedures, such as abdominal and cardiac surgeries, that impair respiratory function and present a high incidence of pulmonary complications [11, 12]; however, two systematic reviews, in 1994 and 2011, showed that there are few studies to support the use of intermittent positive airway pressure aiming to recover lung function and prevent pulmonary complications after major surgery [13, 14]. Recently, clinical trials enrolling patients undergoing lung resection have shown that continuous positive airway pressure (CPAP) is able to improve clinical outcomes including functional capacity, dyspnoea, and oxygenation [15, 16].

The contradiction in the literature and the need for evidence-based practice, focus attention on the following aspects: the results of clinical trials with good methodological quality, the clinical experience of the physiotherapist, and the patient’s preferences [17]. The aim of this systematic review is to provide, through rigorous technical analysis of the existing literature, well-founded advice to help health professionals choose the best treatment for the care of patients undergoing thoracic procedures.

This systematic review will answer the following questions:

- Does non-invasive positive airway pressure reduce the incidence of pulmonary complications, the need for invasive mechanical ventilation, and the mortality rate in patients undergoing thoracic procedures?
- Does non-invasive positive airway pressure improve/recover lung volumes and oxygenation in patients undergoing thoracic procedures?
- Does non-invasive positive airway pressure reduce the time of hospital stay in patients undergoing thoracic procedures?
- Does non-invasive positive airway pressure cause pleural fistulae, pneumothorax, or aerophagia following thoracic procedures?

## Methods/design

### Design of the study

Systematic review

### The inclusion criteria for the studies in this review

#### Types of studies

Randomized and quasi-randomized controlled trials are eligible for this review.

#### Types of participants

The eligible participants will be adults (at least 18 years old) who have undergone a thoracic procedure, defined as any invasive procedure performed in the chest wall.

#### Types of interventions

Use of CPAP, Bilevel Positive Airway Pressure (BiPAP), and Intermittent Positive Airway Pressure Breathing (IPPB) compared with sham device or other types of devices that generate positive pressure or other lung expansion techniques or no intervention.

#### Outcomes

1. The rate of pulmonary complications (as defined by individual studies)
2. The pulmonary volumes assessed by pulmonary function (complete or spirometry)
3. The blood oxygenation assessed by pulse oximetry or arterial blood gas analysis
4. The duration of the hospital stay
5. The rate of tracheal intubation needed for invasive mechanical ventilation
6. The mortality rate
7. Adverse effect as pleural fistula, pneumothorax, aerophagia or other harm resulting from positive pressure interventions

#### Search strategy

The following databases will be searched for all of the available years: Medline, CINAHL, AMED, PsycINFO, Pubmed, LILACS, SciELO, Scopus, PEDro, and the Cochrane Library (specifically, the Cochrane Database of Systematic Reviews and the Cochrane Central Register of Clinical Trials). The search will not be limited by date, language, or publication status. We will check the list of references of any eligible study identified to search for additional relevant studies.

#### Search terms

Terms related to thoracic procedures and modalities of non-invasive positive airway pressure will be used. Search terms are shown in Additional file 1. Two authors (ES and LT) will independently review all of the potential studies for inclusion through the preestablished criteria. They will examine the title and abstract and, where necessary, the full text of the studies to determine their eligibility for inclusion. If the authors do not reach an agreement by discussion, a third author (AL) will make the final decision on eligibility.

#### Data extraction

Two authors (ES and LT) will independently use a standardized method to extract the data from the studies. The discrepancies will be checked against the original data. A third author (AL) will make the final decision in cases of discrepancies.

Data extracted from the studies will be summarized in tables: patients’ characteristics, objectives, type of thoracic procedure, protocol of intervention on experimental and comparative groups, outcome measures, and study results. Adverse effects will also be reported.

#### Quality analysis of the studies

The methodological quality will be assessed using the PEDro scale [18] by a trained author (ES) [19]. The strength of the recommendations will be summarized using the GRADE scale [20]. The rating will be based on available information from the published version and communication with the authors. No eligible study will be excluded on the basis of low methodological quality.

We will also present tables summarizing risk of bias for each study and main biases identified for each study on design and outcomes analysis. Information about the strength of the recommendations for every intervention (CPAP, BiPAP, and IPPB) on each type of thoracic procedure (lung and pleura) will be reported.

### Meta-analysis

For the binary outcome measures (the dichotomous variables), we aim to calculate a pooled estimate of the treatment effect using the risk ratio and a confidence interval (CI) of 95 %. For the continuous outcomes measures, we will calculate a pooled estimate of the treatment effect through the mean difference of the calculation and the 95 % CI. In case of a lack of data, incomplete data or inaccurate data, we intend to contact the researchers responsible for the studies. If we fail to obtain the necessary data for the analysis, we will outline the text of the study results.

Heterogeneity among the studies will be assessed by using *I*^2^ statistic. The values of *I*^2^ range from 0 to 100 %. A value less than 25 % indicates low heterogeneity, values between 25 and 50 % indicate moderate heterogeneity, and a value over 50 % indicates high heterogeneity among the studies [21]. In case of high heterogeneity and sufficient studies included in the review, we will search the possible causes performing the subgroup analyses. Potential subgroups are type of procedures (lung and pleura); type of intervention on comparative groups and intensity (number and duration of sections) of treatment application.

Forest plots will be used to report effect estimates and confidence intervals focusing on meta-analysis and subgroup analysis when possible [21].

### Data reporting

Findings will be reported according to the preferred reporting items for systematic reviews and meta-analyses (PRISMA) as shown in the Additional file 2 [22].

## Discussion

This review aims to provide the best available evidence on the effects of non-invasive positive airway pressure in the incidence of pulmonary complications, lung volumes, blood oxygenation, the need for invasive mechanical ventilation, and the incidence of mortality in patients undergoing thoracic procedures. This evidence will inform physiotherapists, respiratory therapists, surgeons, other healthcare professionals, and patients about the value of these interventions.

## Additional files

Additional file 1:
**Search terms.** Terms that will be used to search articles in databases. Additional file 2:
**PRISMA checklist.** Items that will be described in the report of this systematic review.

## References

[1] Brooks-Brunn J (1995). Postoperative atelectasis and pneumonia: risk factors. Am J Crit Care.

[2] Tulla H, Takala J, Alhava E, Hendolin H, Manninen H, Kari A (1995). Breathing pattern and gas-exchange in emergency and elective abdominal surgical patients. Intensive Care Med.

[3] Reeve JC, Nicol K, Stiller K, McPherson KM, Deneh L (2008). Does physiotherapy reduce the incidence of postoperative complications in patients following pulmonary resection via thoracotomy? A protocol for a randomised controlled trial. J Cardiothorac Surg.

[4] Kroenke K, Lawrence VA, Theroux JF, Tuley MR, Hilsenbeck S (1993). Postoperative complications after thoracic and major abdominal surgery in patients with and without obstructive lung disease. Chest.

[5] Sarkar P, Chandak T, Shah R, Talwar A (2010). Diagnosis and management bronchopleural fistula. Indian J Chest Dis Allied Sci.

[6] McManigle JE, Fletcher GL, Tenholder MF (1990). Bronchoscopy in the management of bronchopleural fistula. Chest.

[7] Pasquina P, Tramèr MR, Walder B (2003). Prophylactic respiratory physiotherapy after cardiac surgery: systematic review. BMJ.

[8] Ott J, Keilty S, Noone L (1992). Intermittent positive pressure breathing—a dying art?. Physiotherapy.

[9] Müller AP, Olandoski M, Macedo R, Costantini C, Guarita-Souza LC (2006). Comparative study between intermittent (Müller Reanimator) and continuous positive airway pressure in the postoperative period of coronary artery bypass grafting. Arq Bras Cardiol.

[10] Lima VP, Bonfim D, Risso TT, Paisani DM, Junior JFF, Chiavegato LD (2008). Influence of pleural drainage on postoperative pain, vital capacity and six-minute walk test after pulmonary resection. J Bras Pneumol.

[11] Awdeh H, Kassak K, Sfeir P, Hatoum H, Bitar H, Husari A (2015). The SF-36 and 6-minute walk test are significant predictors of complications after major surgery. World J Surg.

[12] da Conceição Dos Santos E, Lunardi AC (2015). Efficacy of the addition of positive airway pressure to conventional chest physiotherapy in resolution of pleural effusion after drainage: protocol for a randomised controlled trial. J Physiother.

[13] Thomas JA, McIntosh JM (1994). Are incentive spirometry, intermittent positive pressure breathing, and deep breathing exercises effective in the prevention of postoperative pulmonary complications after upper abdominal surgery? A systematic overview and meta-analysis. Phys Ther.

[14] Chiumello D, Chevallard G, Gregoretti C (2011). Non-invasive ventilation in postoperative patients: a systematic review. Intensive Care Med.

[15] Nery FP, Lopes AJ, Domingos DN, Cunha RF, Peixoto MG, Higa C (2011). CPAP increases 6-minute walk distance after lung resection surgery. Respir Care.

[16] Ldos Roceto S, Galhardo FD, Saad IA, Toro IF (2014). Continuous positive airway pressure (CPAP) after lung resection: a randomized clinical trial. Sao Paulo Med J.

[17] Hebert R, Jamtvedt G, Hagen KB, Mead J (2011). Practical Evidence-Based Physiotherapy.

[18] Mayer CG, Sherrington C, Herbert RD, Moseley AM, Elkins M (2003). Reliability of the PEDro scale for rating quality of randomized controlled trials. Physical Therapy.

[19] Morton NA (2009). The PEDro scale is a valid measure of the methodological quality of clinical trials: a demographic study. Aust J Physiother.

[20] Balshema H, Helfanda M, Schünemannc HJ, Oxmand AD, Kunze R, Brozekc J (2011). GRADE guidelines: 3. Rating the quality of evidence. J Clin Epidemiol..

[21] Higgins J, Green S(e). Cochrane Handbook for Systematic Reviews of Interventions Version 5.1.0, The Cochrane Collaboration. 2011. http://www.cochrane-handbook.org.

[22] Moher D, Altman DG, Liberati A, Tetzlaff J (2011). PRISMA statement. Epidemiology..

