# Supplementary material for: The effect of non-invasive positive airway pressure therapy following thoracic surgical procedures: protocol for a systematic review
Source: Syst Rev. 2015 Jun 12;4:83. doi: 10.1186/s13643-015-0073-8 (PMC4465161; doi:10.1186/s13643-015-0073-8)
Supplement: Additional file 1: — Search terms. Terms that will be used to search articles in databases. [file 13643_2015_73_MOESM1_ESM.docx]

**Additional file 1**

*Search terms:*

(CPAP OR Continuous Positive Airway Pressure OR BIPAP OR Bilevel OR Bilevel Positive Airway Pressure OR IPPB OR Intermittent Positive Airway Pressure Breathing OR Intermittent Positive Pressure Breathing OR Non-invasive Positive Pressure OR Noninvasive Positive Pressure OR Non Invasive Positive Pressure OR Non-invasive Ventilation OR Noninvasive Ventilation OR Non Invasive Ventilation OR Intermittent Positive Pressure OR Intermittent Positive Pressure Ventilation OR Intermittent Positive Pressure Hyperventilation) AND (Thoracoscopy OR Thoracoscopy Pleural OR Thoracoscopies OR Pleural Endoscopy OR Pleuroscopy OR Pleuroscopies OR Thoracoscopic Surgery OR Thoracoscopic Surgical Procedures OR Thoracoscopic Surgeries OR Thoracotomy OR Thoracotomies OR Thoracostomy OR Video-Assisted Thoracic Surgery OR VATS OR Thoracic Surgery OR Thoracic Surgery Procedure OR Thoracic Surgical Procedures OR Pulmonary Surgical Procedures OR Lung Transplantation OR Lung-heart Transplantation OR Thoracoplasty OR Pleural Drainage OR Thoracic Drainage OR Chest Drainage OR Chest Tube OR Thoracocentesis OR Thoracic Puncture OR Chest Puncture OR Pleural Puncture OR Pneumonectomy OR Pneumonectomies OR Lung Volume Reduction OR Total Lung Lobectomy OR Lung Lobectomy OR Lobectomy OR Segmentectomy OR Lung Segmentectomy OR Bronchoscopy OR Bronchoscopic Surgical Procedure OR Bronchoscopic Surgery OR Mediastinoscopy OR Mediastinoscopy Surgery OR Tracheostomy OR Pleural Biopsy OR Lung Biopsy OR Open Lung Biopsy OR Percutaneous Lung Biopsy OR Surgical Lung Biopsy)
